# *Echinococcus multilocularis* Calreticulin Interferes with C1q-Mediated Complement Activation

**DOI:** 10.3390/tropicalmed8010047

**Published:** 2023-01-07

**Authors:** Siqi Xian, Lujuan Chen, Yan Yan, Jianfang Chen, Guixia Yu, Yuxiao Shao, Bin Zhan, Yanhai Wang, Limei Zhao

**Affiliations:** 1 Department of Pathogenic Biology, School of Basic Medical Sciences and Forensic Medicine, Baotou Medical College, Baotou 014040, China; 2 Department of Pediatrics, National School of Tropical Medicine, Baylor College of Medicine, Houston, TX 77030, USA; 3 Parasitology Research Laboratory, School of Life Sciences, Xiamen University, Xiamen 361102, China

**Keywords:** *Echinococcus multilocularis*, immune evasion, complement C1q, classical complement activation, calreticulin, mast cell

## Abstract

As a zoonotic disease caused by *Echinococcus multilocularis* larvae, alveolar echinococcosis (AE) is one of the most severe forms of parasitic infection. Over a long evolutional process *E. multilocularis* has developed complex strategies to escape host immune attack and survive within a host. However, the mechanisms underlying immune evasion remain unclear. Here we investigated the binding activity of *E. multilocularis* calreticulin (*Em*CRT), a highly conserved Ca^2+^-binding protein, to human complement C1q and its ability to inhibit classical complement activation. ELISA, Far Western blotting and immunoprecipitation results demonstrated that both recombinant and natural *Em*CRTs bound to human C1q, and the interaction of recombinant *Em*CRT (r*Em*CRT) inhibited C1q binding to IgM. Consequently, r*Em*CRT inhibited classical complement activation manifested as decreasing C4/C3 depositions and antibody-sensitized cell lysis. Moreover, r*Em*CRT binding to C1q suppressed C1q binding to human mast cell, HMC-1, resulting in reduced C1q-induced mast cell chemotaxis. According to these results, *E. multilocularis* expresses *Em*CRT to interfere with C1q-mediated complement activation and C1q-dependent non-complement activation of immune cells, possibly as an immune evasion strategy of the parasite in the host.

## 1. Introduction

Echinococcosis is a serious zoonotic disease caused by the infections of *Echinococcus multilocularis* (*Em*) and *Echinococcus granulosus* (*Eg*) larvae, causing alveolar echinococcosis (AE) and cystic echinococcosis (CE), respectively. Of these, AE is more serious and a life-threatening disease with high mortality and poor prognosis if not well treated [1]. The main prevalence of AE is in regions of the Northern Hemisphere, such as Asia, Europe and North America. There are an estimated 18,235 new AE infections or cases each year worldwide [2]. It is considered to be one of the most deadly helminthic diseases in humans [3]. Human AE infection is caused by the accidental ingestion of food contaminated with eggs, which form microcystic metacestode vesicles in the liver [4]. The vesicles remain indefinitely proliferative in infected livers, like a tumor, invading surrounding tissues or even metastasizing to distant organs, such as the spleen and brain. *E. multilocularis* metacestode vesicles that are exposed to host immune system [5] have developed sophisticated strategies to evade host immune responses, including innate and adaptive immunity. Understanding of the parasite-developed immune evasion mechanisms would facilitate the identification of vaccine and drug targets to control *E. multilocularis* and other helminth infections.

The complement system acts as the primary line of defense against invading pathogens and plays a crucial role in the innate and acquired immune responses [6,7]. Many pathogens have developed sophisticated strategies to avoid host complement attack [8,9]. For instance, influenza A virus produces matrix (M1) protein [10], *Borrelia burgdorferi* produces lipoprotein BBK32 [11], *Streptococcus pneumoniae* produces endopeptidase O (PepO) [12] and *Trichinella spiralis* secretes paramyosin [13] to bind with human complement C1q, leading to the inhibition of classical complement activation and the membrane attack complex (MAC) formation on these pathogens. The complement-involved lysis and killing of protoscoleces of *E. multilocularis* has been observed in vitro and in vivo to be abolished by heating serum at 56 °C, or by the depletion of complement with EDTA or cobra venom factor [5,14,15]. However, little is known about the exact molecular mechanism behind the evasion of complement attack developed by *E. multilocularis*.

C1q is the initiator of the classical pathway and activated by the antigen/antibody complex, eventually forming MAC on the surface of pathogens leading to cell lysis and death [16]. In addition, C1q plays an important role in the activation of non-complement functions [17], such as binding to C1q receptors on various immune cells including neutrophils [18], eosinophils [19], mast cells [20,21] and macrophages [22] to induce their recruitment to the infected site and stimulate their ability of adhesion and phagocytosis on invaded pathogens.

Recent studies have identified that helminth-produced calreticulin (CRT) is involved in regulating the host immune system through binding to human complement C1q. CRT is a well-conserved Ca^2+^-binding protein present in the endoplasmic reticulum of all kinds of cells among parasites [23,24]. Some parasite CRTs can inhibit C1q-dependent complement classical pathway activation by binding to the complement component C1q. The intracellular protozoa *Trypanosoma cruzi*, nematode *Brugia malayi*, *Haemonchus contortus* and *Necator americanus* produce calreticulin to interact with C1q and inhibit the activation of classical complement as an immune evasion strategy [25,26,27,28]. However, whether tapeworm CRT is involved in host complement activation has not yet been investigated. In our previous research, we provided evidence that *E. multilocularis* calreticulin (*Em*CRT) is expressed on the surface of *E. multilocularis* larvae as well as in the secreted products of protoscoleces (PSCs) and metacestode vesicles. Mice immunized with recombinant *Em*CRT achieved protective immunity against *E. multilocularis* infection [29], indicating its potential as a vaccine candidate. In the current study, we investigated whether *Em*CRT bound to human complement C1q that was involved in the protective immunity against host complement attack. Our results revealed that *Em*CRT had the ability to bind to human C1q, which inhibited the activation of classical complement and C1q-mediated mast cell migration, indicating that *E. multilocularis*-expressed CRT may be involved in the survival strategy of parasite in the host by escaping complement attack.

## 2. Materials and Methods

### 2.1. Animals

Female Kunming mice aged 6–8 weeks were purchased from Xiamen University Laboratory Animals Center (XMULAC) and raised at Xiamen University Animal Facility with free access to food and water. All animal protocols were approved by the Institutional Animal Care and Use Committee of Xiamen University (approval number: 2013-0053) and abided by the NIH Guidelines for the Care and Use of Laboratory Animals.

### 2.2. Sera

After informed consent was obtained, normal human sera (NHS) were collected from the blood of eight healthy donors. The Institutional Review Board (IRB) of Baotou Medical College approved the project with approval number: 2018026. The human serum deficient in C1q (C1qD) was purchased from Merck (Darmstadt, Germany).

### 2.3. Parasites, Antigen and Recombinant Protein Preparations

*E. multilocularis* used in this study was originally obtained from an infected fox in Hulunbeier Pasture of Inner Mongolia, China [30], and maintained in Kunming mice as metacestode vesicles as described [31]. The crude somatic extracts of protoscoleces (PSCs) and metacestode vesicles, and vesicle fluid protein were obtained according to our previously established protocol [29]. His-tagged recombinant *Em*CRT (r*Em*CRT) was expressed in *E. coli* BL21 under induction of 0.4 mM IPTG and purified by Ni-affinity chromatography (Beyotime Biotechnology, Shanghai, China) as described [29].

### 2.4. Cell Culture

HMC-1, the human mast cell line obtained from Qingqi (Shanghai Biotechnology Development Co., Ltd..., Shanghai, China), was grown in DMEM medium containing 1 × streptomycin, penicillin and amphotericin B and 10% FBS in 5% CO_2_ at 37 °C.

### 2.5. Binding of EmCRT to Human C1q

To determine the binding activities of *Em*CRT to human complement C1q, the following immunological assays were performed:

ELISA: A 96-well plate was coated with various quantities of human complement C1q (0, 0.2, 0.4, 0.6, 0.8, 1.0, 1.2 and 1.5 μg in 100 µL coating buffer) (Merck, Darmstadt, Germany) and diluted in 100 μL of pH 9.6 coating buffer for each well overnight at 4 °C. In control plates, each well was coated with the same amount of bovine serum albumin (BSA) (Solarbio, Beijing, China). After being washed three times with PBS + 0.05% Tween-20 (PBST), the plate was blocked with BSA in PBS at 37 °C for one hour. Then, the plate was incubated with different amounts of r*Em*CRT (0, 0.2, 0.4, 1.2, 2 μg/well) in 100 μL of binding buffer (20 mM Tris-HCl, pH 7.4, 1 mM CaCl_2_ and 50 mM NaCl) overnight in refrigerator. The r*Em*CRT bound to human C1q was measured using mouse anti-His mAb (BOSTER Biological Technology Co., Ltd..., Chengmai, China) with 1:3000 dilutions in PBS and 1:5000 HRP-conjugated goat anti-mouse IgG (BOSTER Biological Technology Co., Ltd..., Chengmai, China). The substrate o-phenylendiamine dihydrochloride (OPD, Beyotime Biotechnology, Shanghai, China) was added and OD_450_ was measured with an ELISA reader (Thermo, Waltham, MA, USA).

Far Western blotting: The same amount of C1q (5 μg) and BSA (5 μg) were separated in 15% polyacrylamide gel and transferred onto PVDF membrane (Merck, Darmstadt, Germany). After being blocked with 3% BSA, the membrane was incubated with r*Em*CRT at 5 μg/mL in binding buffer at 37 °C for two hours. The binding of r*Em*CRT to C1q was detected with the same antibodies as mentioned above.

Immunoprecipitation: To further determine whether C1q was able to bind to non-denatured r*Em*CRT or native *Em*CRT derived from different stages of worm extracts, Protein A + G Agarose (Thermo, Waltham, MA, USA) were incubated with 5 µg of anti-His antibody + 5 µg r*Em*CRT, or 5 µL anti-*Em*CRT antisera + crude extracts of metacestode vesicles, PSCs or metacestode vesicle fluid (each 20 µg) overnight at 4 °C. Total 10 µg of human C1q was added to incubate overnight at 4 °C. After being centrifuged at 1000× *g* at 4 °C for 3 min, the supernatant was discarded and the agarose bead–protein complex was washed three times with radioimmunoprecipitation assay (RIPA) lysis buffer (BOSTER Biological Technology Co., Ltd..., Chengmai, China). Finally, 30 µL RIPA buffer was added and the protein complex in the supernatant was separated by SDS-PAGE and then transferred onto PVDF membrane. The C1q pulled down by the non-denatured recombinant and native *Em*CRT protein was determined using rabbit anti-human C1qA antibody (Abcam, Cambridge, UK) at 1:4000 dilutions.

### 2.6. C3 and C4 Deposition Assay

As a C1q activator, human IgM (Sigma, St. Louis, MO, USA) was coated on 96-well plates at 2 µg/mL overnight at 4 °C, then blocked with 5% BSA in PBS at 37 °C for 2 h. On each well of the plates, 2 µg of C1q was added that had been pre-incubated with different amounts of r*Em*CRT (0, 2, 4 µg) or BSA (4 µg, as a comparison) in total volume of 100 µL for 2 h at 37 °C and incubated for 1 h at 37 °C. After washing with PBST, C1q-D diluted at 1:100 in 1 × Veronal buffer (VB, Lonza, Basel, Switzerland) containing 0.05% Tween-20 and 0.1% gelatin was added into each well for 1 h at 37 °C to finish the classical complement activation. NHS at dilution of 1:50 was used as a positive control. After being washed for three times with PBST, each well was added with 100 µL goat anti-human C4 mAb (1:1000 Abcam, Cambridge, UK) or rabbit anti-human C3 polyclonal antibodies (1:100, BOSTER Biological Technology Co., Ltd..., Chengmai, China) to determine C4 and C3 intermediate product of classical complement activation. HRP-conjugated rabbit anti-goat or goat anti-rabbit IgG (1:5000 or 1:1000, Affinity Biosciences, Liyang, China) was used as the secondary antibody and OPD (Beyotime Biotechnology, Shanghai, China) was used as the substrate.

### 2.7. Hemolytic Assays

To determine whether r*Em*CRT inhibited classical complement-activation-mediated hemolysis of sheep red blood cells (SRBC), fresh SRBC at 5×10^8^ cells/mL in 1×HBSS^++^ (Hank’s balanced salt solution supplemented with 0.15 mM CaCl_2_ and 1 mM MgCl_2_, Solarbio, Beijing, China) was sensitized with rabbit anti-erythrocyte antibody (Zhengzhou Baiji Biotechnology Co., Ltd..., Zhengzhou, China) at 37 °C for 30 min. After being washed with 1×HBSS^++^, the antibody-sensitized SRBC were incubated with 1 µg of C1q pre-incubated with 0, 1, 2 or 4 µg of r*Em*CRT or 4 ug or BSA in total volume of 100 µL followed by adding C1q-D (8% in HBSS^++^) for 1 h at 37 °C to complete the complement classical activation. The reaction was stopped by adding cold HBSS^++^ containing 10 mM EDTA. The supernatants were collected by centrifuging at 1200× *g* for 10 min, and OD_412_ was measured. The erythrocyte lysis rate was calculated with total hemolysis in water as control.

### 2.8. Inhibition of rEmCRT on the Binding of C1q to IgM

To determine whether r*Em*CRT inhibited C1q binding to IgM, the plates were coated with human IgM (2 µg/mL) and then blocked with 2% BSA in PBS for 2 h at 37 °C. C1q (1 μg) was pre-incubated with r*Em*CRT or BSA (0, 0.5, 1, 1.5, 2, 2.5 and 3 μg in 100 µL binding buffer) at 37 °C for two hours. The reaction complex was added to the IgM-coated plates overnight at 4 °C. Anti-C1q polyclonal antibody (Abcam, Cambridge, UK) at 1:1000 dilution was used to detect remaining C1q on IgM-coated plates.

### 2.9. Cell Immunofluorescence Labeling

To evaluate whether r*Em*CRT inhibited C1q binding to the C1q receptor on mast cells, HMC-1 cells were adhered on glass slides, then fixed for 20 min at room temperature with paraformaldehyde 4%. The fixed HMC-1 cells were blocked with normal goat serum (BOSTER Biological Technology Co., Ltd..., Chengmai, China) for 30 min at room temperature. Human C1q at concentration of 80 μg/mL was pre-incubated with different amounts of r*Em*CRT (0, 30, 60 and 80 μg/mL) for one hour at 37 °C, then transferred to the HMC-1 cells on the slides. Rabbit anti-C1q mAb (Abcam, Cambridge, UK) diluted at 1:100 in PBS was used to measure the binding of C1q to HMC-1 cells. DAPI staining was performed to show nuclei of the cells (Solarbio, Beijing, China). Confocal laser scanning microscope (Nikon, Tokyo, Japan) was used to acquire the images.

### 2.10. Transwell Chemotaxis Assay

The inhibitory effect of r*Em*CRT on the C1q-induced chemotactic migration of HMC-1 cells was identified using an 8-µm-pore transwell chamber (Corning, New York, NY, USA). Total 200 μL of DMEM medium containing 2% FBS was added to the upper chamber with 3 × 10^5^ HMC-1 cells. The 10 nM human C1q was pre-incubated with various concentrations of r*Em*CRT (0, 3, 6 μg) in 500 μL DMEM medium with 5% FBS, then transferred to the lower chamber to initiate chemotactic migration of HMC-1 cells at 5% CO_2_, 37 °C for 24 h. The cells that migrated through the membrane to the lower chamber were counted using a flow cytometer (Beckman Coulter, Brea, CA, USA) [21]. LPS (100 ng/mL) was used as a positive control and BSA (6 μg/0.5 mL) as a negative control for the chemotaxis assay.

### 2.11. Statistical Analysis

All data were shown as mean ± standard deviation and one-way ANOVA was performed. All statistical analysis was performed by GraphPad Prism 7 (San Diego, CA, USA). *p* < 0.05 was considered as statistically significance.

## 3. Results

### 3.1. Recombinant EmCRT Binds to Human C1q

**Figure 1:**
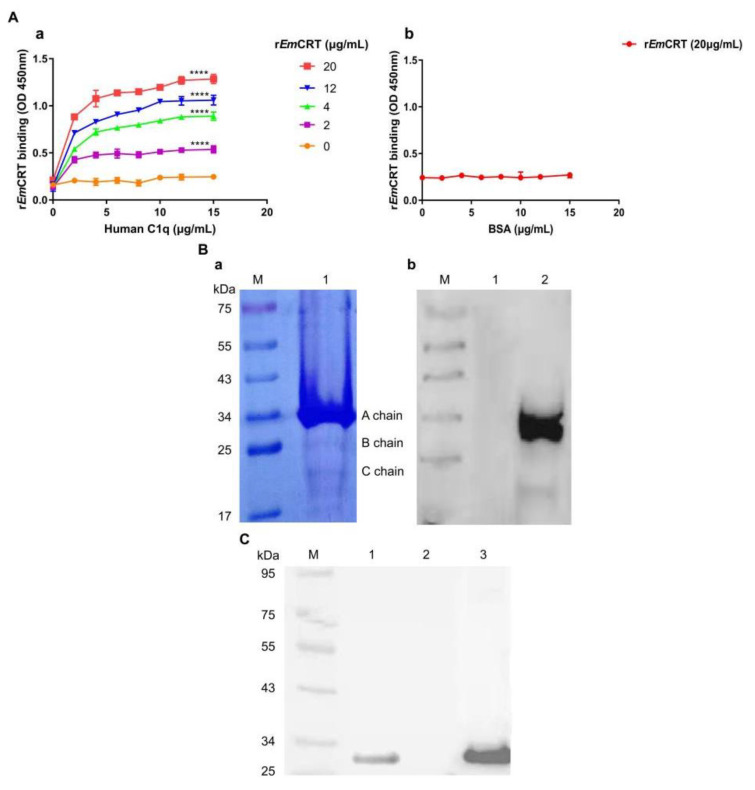
Recombinant *Em*CRT bound to human C1q. (**A**) r*Em*CRT (0, 2, 4, 12, 20 μg/mL) in binding buffer was added to the plate coated with C1q (0, 2, 4, 6, 8, 10, 12 and 15 μg/mL) (**a**) and to BSA-coated plate (20 µg/mL) (**b**), anti-His antibody was used to detect the binding. Data are expressed as the mean ± SDs for three independent experiments (**** *p* < 0.0001 compared to the plate without the addition of r*Em*CRT). (**B**) An amount of 5 µg of C1q complement was separated by SDS-PAGE under reduced condition showing A, B, C chains (**a**). Western blot with anti-His antibody (1:5000) to detect r*Em*CRT binding to C1q (Lane 2), but not to BSA (Lane 1), under reducing condition (**b**). (**C**) Immunoprecipitation showing that C1q was pulled down by r*Em*CRT bound to anti-His antibody/Protein A + G Agarose. Rabbit anti-C1qA antibody was used to detect the eluted complexes. Lane 1, r*Em*CRT + anti-His + C1q; Lane 2, anti-His + C1q; Lane 3, C1q alone.

The interaction between r*Em*CRT and human complement C1q was determined by different immunological assays. ELISA results demonstrated that r*Em*CRT was capable of binding to C1q in a dose-dependent way (Figure 1A, a). Under the same conditions, there was no apparent binding of r*Em*CRT to the BSA-coated plate (Figure 1A, b). SDS-PAGE separation results showed that C1q contained mainly A, and weak B and C chains under reduced condition (Figure 1B, a). The Far Western blotting demonstrated that r*Em*CRT mainly bound to A chain, weakly bound to C chain of complement C1q, but did not bind to BSA as the non-relative control (Figure 1B, b). Further, to confirm whether recombinant *Em*CRT bound to human C1q under non-denatured condition, the natural form of C1q was pulled down by r*Em*CRT bound to anti-His antibody (Figure 1C). Anti-His antibody alone without r*Em*CRT could not pull down C1q. These findings demonstrated that r*Em*CRT was able to bind to the A and C chains of complement C1q.

### 3.2. Native EmCRT from Worm Extracts Binds to Human C1q

**Figure 2:**
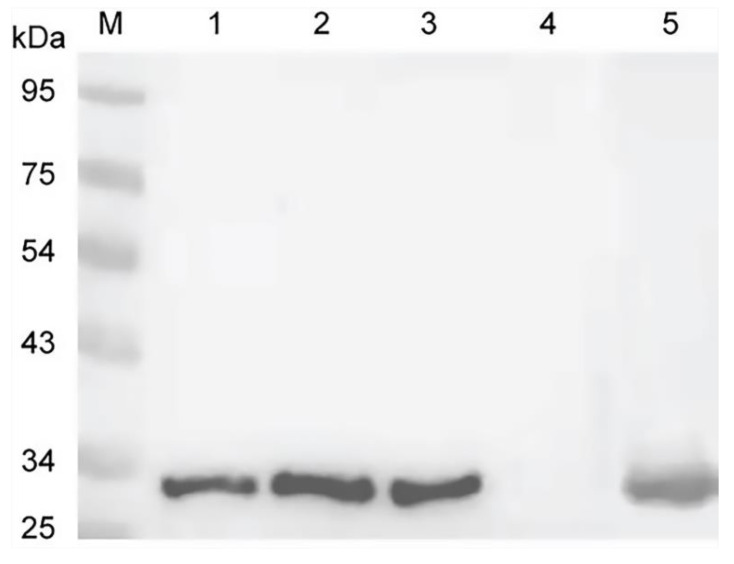
Immunoprecipitation showing the human C1q was pulled down by the native *Em*CRT from worm extracts or vesicle fluid and detected by rabbit anti-C1qA antibody: M, standard protein marker; Lane 1, extracts of metacestode vesicles + anti-*Em*CRT + C1q; Lane 2, metacestode vesicle fluid + anti-*Em*CRT + C1q; Lane 3, extracts of PSCs + anti-*Em*CRT + C1q; Lane 4, anti-*Em*CRT + C1q; Lane 5, human C1q alone (5 µg).

Immunoprecipitation and Western blotting were performed to investigate the binding of native *Em*CRT derived from *E. multilocularis* larval stages to human C1q (Figure 2). The results distinctly showed that C1q could bind to native *Em*CRT derived from worm extracts or vesicle fluid, pulled down by the anti-*Em*CRT polyclonal antibody and detected by anti-C1q antibody. No C1q was seen in the anti-*Em*CRT alone control, suggesting that C1q specifically binds to native *Em*CRT derived from worm extracts or fluid.

### 3.3. rEmCRT Inhibits the Classical Complement Activation Pathway and Hemolysis

**Figure 3:**
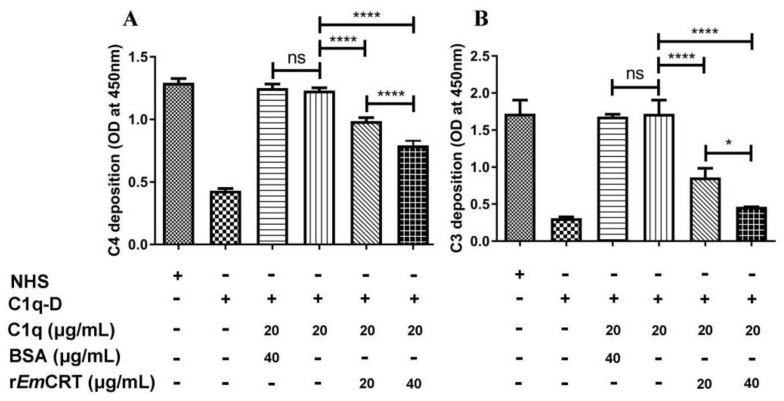
Inhibition of C1q-mediated C4/C3 generation by r*Em*CRT. C1q (20 µg/mL) was pre-incubated with r*Em*CRT (0, 20 or 40 µg/mL) or BSA, (40 µg/mL), then transferred into human IgM-coated plates (2 µg/mL). After being washed, C1q-D serum was added as a supplement of other complement components to trigger classical pathway activation. Anti-C4 or -C3 antibodies were used to detect the deposits of C4 and C3 on the plates. The results are shown as the means ± SDs for three independent experiments. * *p* < 0.05, **** *p* < 0.0001. ns, no significant difference.

To evaluate whether the binding of r*Em*CRT to C1q interferes with C1q-initiated classical complement activation, the activation intermediate products C4 and C3 were measured. The results displayed that classical complement activation can be completed in C1q-D serum by supplementing with C1q at a similar level to NHS based on the levels of C4 or C3 deposited in the plates. However, the addition of r*Em*CRT to C1q significantly decreased C4 and C3 deposition in a dose-dependent way (Figure 3), indicating that the binding of r*Em*CRT to C1q interfered with C1q/IgM-initiated classical complement activation. There was no inhibitory effect in the presence of BSA as a control.

**Figure 4:**
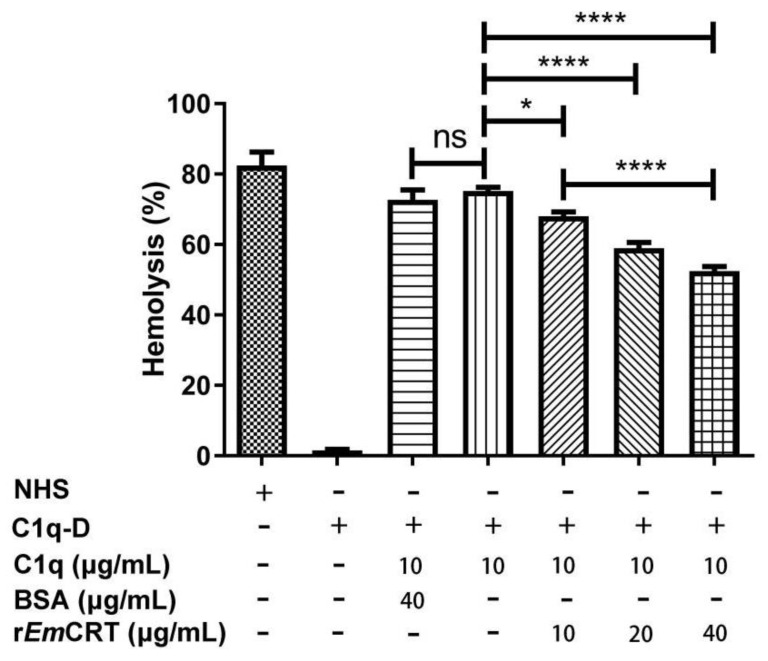
Complement-mediated hemolysis was inhibited by r*Em*CRT. C1q-mediated antibody-sensitized sheep blood cells hemolysis was inhibited by r*Em*CRT in a dose-dependent manner. Three independent experiments were conducted and the results were presented as means ± SDs. * *p* < 0.05 and **** *p* < 0.0001. ns, no significant difference.

To further determine whether r*Em*CRT inhibits C1q-dependent classical pathway, antibody-sensitized SRBC were incubated with C1q pre-incubated with different amounts of r*Em*CRT. As shown in Figure 4, r*Em*CRT inhibited C1q-initiated classical complement-activation-mediated hemolysis in a dose-dependent manner. There was no obvious hemolysis observed in the presence of C1q-D serum alone, since the classical pathway could not be activated in the absence of C1q. BSA as a control protein did not show an inhibitory effect.

### 3.4. rEmCRT Competitively Inhibits the Binding of Human C1q to IgM

**Figure 5:**
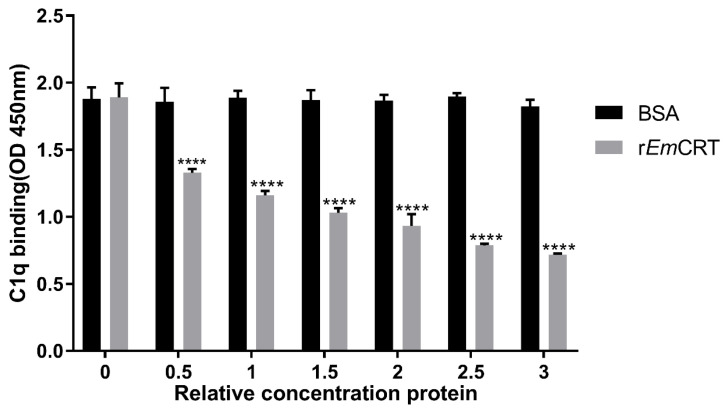
r*Em*CRT inhibited human C1q to bind to IgM. Total 10 μg/mL of C1q was pre-incubated with 0.5 to 3 times excess (*w*:*w*) of r*Em*CRT or BSA and then transferred to the plates coated with IgM. After being washed, anti-C1q polyclonal antibody was used to detect C1q binding to IgM in presence of r*Em*CRT. Data are expressed as mean ± SDs from three independent experiments and statistical analysis was performed using one-way ANOVA. **** *p* < 0.0001 compared to BSA control.

To understand how r*Em*CRT inhibits C1q-mediated classical complement activation, the competitive inhibition assay was carried out in the presence of IgM. C1q was pre-incubated with various amounts of r*Em*CRT before being transferred to IgM-coated plates. The results showed that pre-treatment with r*Em*CRT significantly inhibited C1q’s binding ability to IgM, and the inhibition was dose dependent. No inhibitory effect was observed in BSA control group (Figure 5). The results suggest that r*Em*CRT competes with IgM to bind to C1q.

### 3.5. rEmCRT Inhibits C1q Binding to Mast Cells

**Figure 6:**
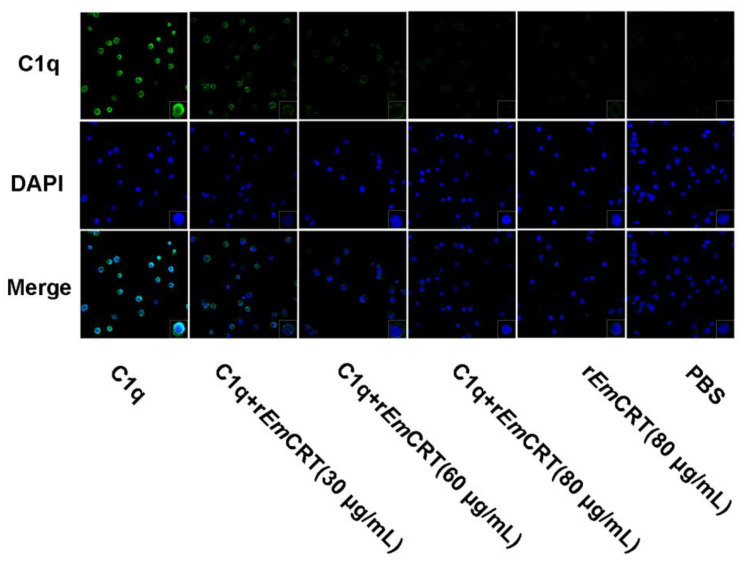
Recombinant *Em*CRT inhibited the binding of C1q to HMC-1 cells. HMC-1 cells were adhered on glass slides and fixed with 4% paraformaldehyde, and incubated with C1q (80 µg/mL) that was pre-incubated with different amounts of r*Em*CRT (0, 30, 60 or 80 µg/mL). The binding of C1q on HMC-1 cells was detected with anti-C1q antibody and FITC-conjugated goat anti-rabbit IgG (green). Nuclei were dyed with DAPI (blue). The magnitude is 400× and one amplified cell located at the lower right corner is 1000×.

To assess whether r*Em*CRT could inhibit the binding of C1q to the C1q receptor on mast cells, C1q (80 μg/mL) was mixed with r*Em*CRT at concentration of 0, 30, 60 and 80 μg/mL. Then, it was transferred into HMC-1 cells. The C1q binding on the mast cells was detected by immunofluorescence staining with anti-C1q antibody. r*Em*CRT inhibited C1q binding to mast cells and the inhibition was dose dependent. No obvious fluorescence was observed in the r*Em*CRT or PBS alone control group (Figure 6). Thus, the results indicate that r*Em*CRT binds to C1q, which interferes with C1q’s binding to mast cells.

### 3.6. rEmCRT Inhibits C1q-Induced Mast Cells Chemotaxis

**Figure 7:**
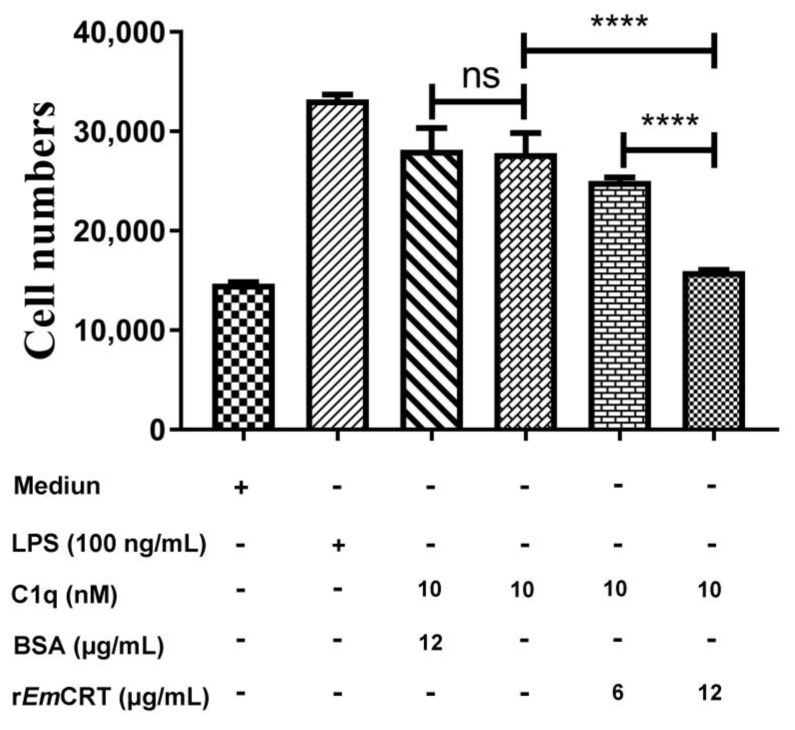
The inhibition of r*Em*CRT on C1q-induced migration of mast cells was conducted in a transwell 24-well plate. In the upper chamber, 3 × 10^5^ HMC-1 cells were seeded per well. An amount of 10 nM of C1q was pre-incubated with r*Em*CRT (0, 6 or 12 μg/mL) and then transferred into the lower chamber. LPS (100 ng/mL) was added as a positive control. The number of cells was counted by a flow cytometer. Data are shown as the mean ± SDs from three independent tests, each test was carried out in triplicate. **** *p* < 0.0001. ns, no significant difference.

To determine the effect of r*Em*CRT on C1q-induced chemotaxis of mast cells, a migration assay using a transwell chamber was conducted. The results revealed that both LPS and C1q significantly attracted HMC-1 cells migration through the membrane (Figure 7). The C1q-induced mast cells migration through the membrane was inhibited by the pre-incubation with r*Em*CRT in a dose-dependent manner (**** *p* < 0.0001). BSA protein had no effect on HMC-1 cells attraction by C1q.

## 4. Discussion

Helminths are multicellular pathogens that generate many macromolecules to regulate host immune responses as an evasion strategy. The complement system is the primary line of immune defense against pathogen invasion, and works by attacking pathogens directly and enhancing and opsonizing the functions of antibodies and immune effectors (neutrophils, eosinophils, mast cells, macrophages, etc.) to eliminate the invaded pathogens [32]. Therefore, blocking complement attack is crucial to pathogens’ survival in the invaded host [6,7,13]. Many pathogens, such as viruses, bacteria and parasites, apparently share comparable strategies or mechanisms to avoid complement attack [9]. However, there is a lack of knowledge about the complement escape mechanism in the parasitism of cestodes.

Calreticulin (CRT) is a well conserved Ca^2+^ binding protein and molecular chaperone. CRT is involved in a spectrum of processes, such as Ca^2+^ homeostasis, antigen presentation and process, cellular adhesion and motility of organisms [33,34]. Available studies demonstrated that CRTs from some parasites, such as protozoa, helminths, and arthropods, have the ability to bind to complement component C1q to interfere with the host complement activation [25]. Furthermore, the binding domain of C1q was located in the S-domain of the protein [35]. In our previous study, sequence alignment reveals that *Em*CRT shares 49–56% sequence identity with other helminth CRTs, including *N. americanus* [28], *H. contortus* [27] and *B. malayi* [26], indicating that it is genetically conserved among helminths [36].

Our previous studies have identified that *Em*CRT was identified on the surface of the *E. multilocularis* and in the excretory-secretory (ES) products of larval stages as well, suggesting its accessibility to host immune systems including complement components, which provides a biological basis to study its interaction with host immune and complement systems. In this study, we demonstrated that both the recombinant *Em*CRT and the natural protein from the parasite were able to bind to C1q, mainly to A chain, indicating the possibility of its function as an inhibitor of classical pathway of complement activation. In fact, we subsequently observed a significant reduction in C4b and C3b intermediate products after C1q was pre-incubated with r*Em*CRT. The antibody-sensitized sheep red blood cells hemolysis was also inhibited as a result of the unsuccessful formation of the membrane attack complex (MAC) due to the binding of *Em*CRT to C1q. Further investigation in this study identified that *Em*CRT competed with IgM to bind to C1q [37]. It explains how *Em*CRT inhibits the IgM-initiated classical complement activation. Except for being involved in the classical complement pathway, C1q also possesses multiple biological functions as a versatile pattern recognition molecule [17]. In response to foreign pathogen invasion, C1q acts as an initiator for classical complement activation and a non-complement immune activator as well. In this study, we investigated the effect of *Em*CRT on the C1q-mediated mast cell activation except for its inhibitory effect on the complement classical activation pathway. Mast cells as innate immune cells take an active part in the innate immune responses to a number of pathogens and enhance the earliest processes in the development of acquired immune responses [1,2,21,38]. During chronic atopic disease or helminthiasis, mature mast cells are infiltrated at the sites of inflammation. Two specific C1q receptors, cC1q-R (binding to the collagen-like stalk of C1q) and gC1q-R (binding to the globular heads of C1q), were found on mast cells. C1q binds to the C1q receptors on the mast cells to act as an attractant to induce mast cells migration to infectious or inflammatory sites [21]. In the present study, we demonstrated that C1q could bind to the surface of HMC-1 mast cells, the addition of r*Em*CRT decreased the C1q binding to mast cells in a dose-depend manner, possibly through blocking the binding ability of C1q to the C1q receptor on mast cells. The addition of r*Em*CRT also inhibited C1q-induced HMC-1 cells chemotaxis detected by transwell chamber assays. All in vitro experimental results in this study suggest that *E. multilocularis* produces calreticulin to interfere with C1q-initiated classical complement activation and C1q-induced chemotaxis of mast cells, possibly as a survival strategy of the parasite in the host. However, the actual effect of calreticulin on the complement activation on *E. multilocularis* metacestodes in vivo, or on the protoscoleces in vitro, needs to be further investigated.

Combined with our previous study, our results suggest that *E. multilocularis* produces calreticulin during infection to inhibit C1q-mediated activation of the classical complement pathway and C1q-dependent immune cell activation, possibly as a survival strategy in the hostile immune environment of the host. Therefore, *Em*CRT could be a good vaccine candidate and drug target against *E. multilocularis* infection. The actual impact of *Em*CRT on the complement activation on *E. multilocularis* metacestodes in vivo is under investigation.

## Data Availability

Data included in this article are available from the corresponding author upon request.
